# The impact of early life nutrition and housing on growth and reproduction in dairy cattle

**DOI:** 10.1371/journal.pone.0191687

**Published:** 2018-02-14

**Authors:** G. Curtis, C. McGregor Argo, D. Jones, D. Grove-White

**Affiliations:** 1 Department of Obesity and Endocrinology, Institute of Ageing and Chronic Disease, University of Liverpool, Leahurst Campus, Neston, Wirral, United Kingdom; 2 School of Veterinary Medicine, University of Surrey, Guilford, United Kingdom; 3 Institute of Veterinary Science, University of Liverpool, Leahurst Campus, Neston, Wirral, United Kingdom; University of Illinois, UNITED STATES

## Abstract

Contentious issues in calf rearing include milk feeding practices and single versus group housing. The current study was performed on a high producing 170 Holstein cow dairy farm, to investigate the impact of nutrition and housing on growth and reproduction. Heifer calves (*n* = 100) were allocated in birth order to one of two commonly used management strategies. All calves received 3–4 litres of dam specific colostrum within 6 hours of birth. Group A calves were group housed from birth and fed milk replacer (MR) *ad libitum* via a computerised machine utilising a single teat, with weaning commencing at 63 days of age. Group R calves were initially housed in individual pens and received 2.5 litres of MR twice daily via a bucket until 21 days of age when they were group housed and fed 3 litres of MR twice daily via a group trough with weaning commencing at 56 days. From 12 weeks of age onwards, calves in both dietary groups were subject to common nutritional and husbandry protocols. All breeding of heifers was via artificial insemination with no hormonal intervention. Calves were weighed, body condition scored and morphometric measures recorded weekly up till 12 weeks of age then monthly until conception. Pre-weaning growth rates (kg/day) were significantly higher in Group A calves compared to Group R (0.89, 95% CI 0.86–0.93 *vs* 0.57, 95% CI 0.54–0.6 kg/day P < 0.001) with the most marked differences observed during the first three weeks of life (0.72, 95% CI 0.61–0.82 *vs* 0.17, 95% CI 0.08–0.26 P < 0.001). Whilst Group A calves gained body condition score (BCS) throughout the pre-weaning phase, Group R calves lost BCS during the first 4 weeks of life. Data suggested that Group R calves supported skeletal growth during this period by catabolising body tissue. Group A calves had a greater risk of disease than group R calves during the pre-weaning phase (diarrhoea: odds ratio 3.86, 95% CI 1.67–8.9; pneumonia: odds ratio 5.80, 95% CI 2.33–14.44) although no calves died during this period. Whilst pneumonia had a significant impact on growth during the study duration (P = 0.008), this was not the case for diarrhoea. Whilst univariate analysis failed to show any statistically significant group differences (P > 0.050) in any of the mean values of measured reproductive parameters, multivariable Cox regression suggested that there was a weak trend (P = 0.072) for Group A animals to achieve first service earlier than their Group R counterparts (62.6 weeks *versus* 65.3 weeks). Irrespective of dietary group, the hazard for achievement of all measured reproductive parameters, apart from time to puberty, was 20–40% less for heifers borne from multiparous dams compared to heifers from primiparous dams.

## Introduction

Dairy heifer rearing involves significant financial input accounting for approximately 20% of total on farm costs [1–3]. Published data recommend that the optimal age at first calving (AFC) is 24 months [4–6]. Below this age, heifers are considered unlikely to have sufficient body size to support their genetic potential for lifetime milk production or to easily deliver a healthy calf [5]. Conversely, rearing costs will be increased for animals with a greater AFC [7]. Achieving this optimal AFC depends largely on early life management and nutrition [7–10] ensuring that animals are sufficiently well grown to receive their first insemination at 12–13 months. Whilst the optimal AFC is 24 months, there is debate as to the ideal weight and height targets for first service in Holstein heifers, with a paucity of peer reviewed recommendations. This is attributable in part to the increase in mature body size of the Holstein cow over the past 10–20 years, with animals becoming heavier and taller in stature [11]. Current targets commonly used in the UK dairy industry are weight at 1^st^ service of between 380–400 kg and a minimum withers height of 125cm [12].

The provision of restricted amounts of milk or milk replacer (MR) for pre-weaned dairy calves is commonplace on U.K. dairy farms [13], despite concerns that this practice may result in calves receiving a sub-optimal supply of nutrients for growth and development [14,15]. This practice is justified by anecdotal or short-term evidence [16], coupled with the requirement for maximal rumen development at weaning [17]. Studies which evaluated rumen characteristics of veal calves fed large volumes of milk reported minimal rumen development at weaning [18,19]. Early transition from MR to cheaper solid feedstuffs has traditionally been considered an important economic objective for dairy producers. However, restricting MR provision, in order to encourage early transition to solid food, may deprive the calf of sufficient dietary energy to support growth in early life [15]. There is increasing evidence that growth rates and MR intake prior to weaning are positively associated with increased productivity in later life [20], suggesting that restricting MR provision in early life may constrain long term production potential.

Post-weaning, heifers should be provided with a diet that allows growth at a sufficient rate without fattening: a growth rate of between 0.8–0.9 kg per day is considered optimal [21] in terms of body composition and ensuring animals reach an adequate size and weight for service (55% of mature bodyweight) to calve down at 24 months of age. Furthermore, age of onset of puberty has been shown to be affected by plane of nutrition in a study where dairy heifers (aged 4.5–9.5 months) fed an accelerated (1000g/day weight gain) feeding regime reached puberty at least one month earlier than animals fed a standard diet (700g/day weight gain) [22].

The present study compared data from calves subjected to one of two pre-weaning management strategies: 1) restricted milk replacer (twice daily bucket feeding) with individual penning for the first 21 days of life followed by group housing or 2) *ad libitum* access to milk replacer with group housing from birth. The objectives were to describe growth and reproductive parameters of heifers from birth until attainment of first pregnancy.

## Materials and methods

This study was performed in compliance with Home Office (Animal Scientific Procedures Act, 1986) legislation and was approved by the University of Liverpool Animal Welfare Committee. The study was performed between April 2011 and April 2014 at the University of Liverpool’s Wood Park Dairy Farm, Neston, Wirral, U.K. (53°N). All healthy singleton heifer calves born between January 2011 and November 2012 were recruited at birth into either an *ad libitum* (Group A, *n* = 50) or restricted (Group R, *n* = 50) MR feeding cohort for the first 12 weeks of life.

Calves were born into group calving accommodation with between 5 and 15 cows present. If born between 08:00 and 18:00 hours, calves were removed from their mothers within 4 hours of birth and taken to calf accommodation; if born between 18:00 and 08:00 hours, calves remained with their dam for up to 12 hours before being transferred to the calf house. Dam parity was recorded and calves were assigned to one of the two MR feeding strategies on arrival at the calf house in alternate groups of ≤ 6, such that each group or pen of calves had an age range of no more than 14 days (Table 1).

**Table 1:** Nutritional and husbandry protocols used throughout the first 12 weeks of the study. All calves in Group A (*ad libitum* MR access) and Group R (restricted MR access) were fed and housed using the described protocols.

Group	Milk Replacer allowance	Milk Replacer feeding method	Weaning Protocol	Housing Method	Concentrate and Forage
**A**	*Ad libitum* access from birth until day 63	Automatic teat feeder	Stepwise restriction of daily MR allowance over 21 days (0.7L restriction/day). Completed by day 84	Group housed from birth (n≤6)	*Ad libitum* access to grass hay and up to 2.5kg concentrate feed (coarse mix) daily
**R**	5L daily from day 4 until day 21, then 6L daily until day 56 (provided as 2 equal meals, 09:00 & 17:00hrs)	Individual bucket to day 21, thereafter group trough fed	50% reduction of MR allowance over 7 days prior to stopping milk feeding. Completed by day 63	Individually housed until 21 days then group housed (n ≤ 6)	*Ad libitum* access to grass hay and up to 2.5kg concentrate feed (coarse mix) daily

Between 3 and 4 litres of calves own dam’s colostrum (collected as soon as possible after birth) was administered to each calf via an oesophageal feeder at the earliest opportunity after birth. Freshly-collected, dam-specific colostrum meals i.e. “transition milk” were then fed twice daily via individual buckets (2 litres per feed) for four days at which time MR feeding was introduced to Group R calves. The composition of the MR was 96.97% DM, 7% ash, ME 21.570 MJ/kgDM, pH 5.96, 18% Oil, 23% Protein based on fat filled whey protein phospholipid concentrate, with a small proportion of hydrolysed wheat gluten (10%), additives (1.3%), and synthetic amino acids (Volac International Ltd, UK). The specific gravity of the initial colostrum meal was assessed with a Brix refractometer (Animal Reproduction Systems, CA, U.S.A.). For Group A calves, familiarisation and training for use of the automatic computerised teat feeder (Vario feeder, Forster Technik, Germany) from which *ad libitum* MR was dispensed began on entry to the calf house. Calves in this group were able to access MR from birth in addition to the 4 day dam specific colostrum meals. Milk replacer powder was thoroughly mixed with water (125g MR/litre, 37°C) immediately prior to feeding for both dietary groups. The age and timing of weaning from MR differed between the 2 dietary groups. Weaning commenced in Group R calves at 56 days and was completed by day 63, whilst in Group A calves it commenced at 63 days and was completed by day 84 (Table 1).

Milk replacer intakes for individual calves in Group A were recorded daily from the computerised feeder (± 0.1litres). Concentrate feed (Primestart coarse mix, 86.2% DM, 18% crude protein, 8% crude fibre, 9.5% ash, 3.5% oil, ME 14.459 MJ/kgDM, BOCM Pauls Ltd U.K.) was supplied daily on an *ad libitum* basis up to 2.5 kg per head from birth. Individual intakes could not be measured for group penned calves; however mean group intakes were recorded for a subset of calves: 2 pens of Group A calves (*n* = 9) and 2 pens of Group R calves (*n* = 7) between April and September 2012.

### Housing

Calves in Group R were housed individually in metal gated pens (1m x 2m) over raised slatted flooring bedded with wheat straw from birth until 21 days of age. At 21 days of age, Group R calves were moved to deep wheat straw-bedded group pens (5m x 6m, *n* ≤ 6, age range ≤ 14 days). Calves in Group A were grouped by age (range ≤ 14 days, *n* ≤ 6) and were directly introduced to identical group pens on entry into the calf house. All calves had *ad libitum* access to forage (grass hay 85% DM, 8.5% crude protein, 8% crude fibre, 7.4% ash, ME 8.4 MJ/kgDM), fresh water and coarse mix concentrate feed, up to a maximum of 2.5 kg per head daily.

From 12 weeks of age onwards, calves in both dietary groups were subject to common nutritional and husbandry protocols. Irrespective of pre-weaning dietary group, at 4 months of age all calves were transferred to follow-on accommodation (indoor straw yards 18m x 6m, approximately *n* = 12 per group). Calves in the straw yards were fed along the front (18m) of each yard ensuring adequate bunk space. However from 9 months of age until conception was confirmed, heifers were housed in free-stall accommodation with 31 free-stalls (1.93m x 1.14m), total available bunk space/feed barrier was 35.3 metres.

From 3 to 5 months of age, the diet consisted of a maximum of 2.5 kg of concentrate feed per head (86.20% DM, 18.00% crude protein, 4.00% oil, 9.50% ash, 12.50% crude fibre, ME 14.551 MJ/kg Super Rearer 18 nuts, BOCM Pauls, U.K.). Grass hay and fresh water were freely available at all times.

In accordance with standard farm practice, nutrition from 5 months onwards was highly variable consisting largely of refusals from the total mixed rations fed to the lactating, far off dry and transition dry cows. However, depending on the quantity of refusals available each day, additional maize silage or grass silage was added to the diet. Whilst no precise rationing was performed, approximately 12 kg on a fresh weight basis (dry matter ~ 50%) was supplied per head daily. It was not possible to estimate individual feed intakes since animals ranged in body weight from approximately 200–400 kg.

A target body weight of 380kg and withers height of 125cm were set by the farm management as minimum standards which animals were to achieve before first service. All heifers were served via artificial insemination of selected semen following once per day visual heat detection (no synchronisation or other heat detection methods were employed).

### Morphometric measurements

The body weight of each calf was recorded within 12 hours of birth (Ritchey Ltd, North Yorkshire, U.K., ± 0.5kg). During the following 36 hours, measures of the height at the highest point of the withers and loin (±0.1cm, wooden measuring stick, I&D Smallwood, U.K.), circumference of the heart girth (immediately caudal to the elbow) and belly girth (widest part of the belly) (± 1cm), crown to rump length (CRL) (± 1cm) and hock-fetlock length (HFL) (± 1cm, plasticised tape measure) were recorded and body condition score (BCS) was recorded in accordance with the system presented by Edmonson *et al* [23] (1 = emaciated 5 = obese). All body weight, morphometric and BCS measures were repeated weekly to 12 weeks of age, then every 4 weeks until attainment of pregnancy.

### Laboratory measurements

Heparinised blood samples (7ml) were collected from all calves within 48 hours of birth and weekly from 28 weeks of age until puberty was confirmed. Samples were stored on ice prior to harvesting plasma, which was stored at—20°C for up to 3 months pending analyses. Adequacy of passive transfer of Immunoglobulin (Ig) was assessed by measuring calf plasma total protein (PTP) concentrations at 48 hours of age using a refractometer (Clinical refractometer, Hayes, U.K.) taking a cut-off of 56 g/l as indicative of failure of passive transfer (FPT) of Ig [24]. From 28 weeks of age onwards ‘pregnane metabolites’ were measured in triplicate as a proxy for progesterone concentrations using a previously validated ELISA test [25]. The minimum detectable concentration was 0.08 ng/ml, intra and inter-assay variation were 8.3 and 14% respectively. Animals were classified as pubertal when plasma pregnane metabolite concentrations of ≥2.00 ng/ml were recorded for 2 consecutive weeks.

### Health measurements

All pre-weaned calves were examined twice daily for signs of illness by the researchers. Diarrhoea was diagnosed on the basis of a faecal score > 2 [26]. Pneumonia was diagnosed on the basis of the presence of one or more of the following signs, accompanied by a rectal temperature > 39.4° C: nasal discharge, ocular discharge, coughing, increased respiratory rate [26]. Cases of diarrhoea received 2L twice daily of oral rehydration solution (Effydral: Zoetis) by bucket and teat or oesophageal feeder if no suck reflex was present. Rehydration therapy was continued for at least 3 days. Diarrhoeic calves were not removed from their accommodation and continued being offered MR at the same rate as their healthy counterparts, although they did not always consume their full allocation. Cases of pneumonia were treated once with sub-cutaneous injections of tulathromycin (Draxin, Zoetis) at a dose rate of 2.5 mg / kg and Meloxicam (Metacam, Boehringer Ingleheim) at a dose rate of 0.5 mg / kg.

### Statistical analysis

All data were initially entered into an Excel spreadsheet (Microsoft Corp, USA) and exported to STATA 13 (StataCorp, Texas, USA) for analysis.

#### Heifer calves at birth

Simple univariable analyses using linear regression and Students t tests were carried out initially to investigate possible associations between the measured variables. Outcome variables of interest were, birth weight, colostrum quality and plasma total protein concentration at 48 hours.

#### Heifer calves from birth until pregnancy

Daily changes of body weight and other morphometric measures from birth to 108 weeks of age were calculated for discrete time periods throughout the study. Student’s t tests were used to compare the mean measurements at different time points between calves in Group A and Group R.

Random effects linear regression models were then fitted using backward stepwise selection for all morphometric measures from birth until pregnancy. All remaining covariates were included in the initial model. A backwards, stepwise model-building strategy [27] was employed whereby a full model was built and then each variable removed in turn, a likelihood ratio test performed and the resultant P value noted. The variable with the highest P value was then omitted and the process repeated. This process was repeated until only variables with P<0.2 remained in the model. The omitted variables were then added back in turn, starting with the lowest P value, a likelihood ratio test performed after each addition, and the variable retained if P<0.2. This process was continued until no further variables could be added, to produce the final model. Calf identity was included as a random effect to account for clustering in all models.

The following explanatory variables were initially offered to the full body weight model: pre-weaning dietary group (*ad libitum* or restricted MR), age in weeks, an age*diet interaction term, dam parity (primiparous *v* multiparous), plasma TP and occurrence of diarrhoea and/or pneumonia during the first 12 weeks. Other interaction terms were offered if considered biologically feasible and retained if their inclusion improved model fit.

The final explanatory variables for the body weight model were forced into models for the other morphometric measures (withers and loin height, heart and belly girth, CRL, HFL and BCS). Predicted marginal means were estimated from multivariable models and plotted graphically where appropriate.

Survival analysis was employed to assess the impact of pre-weaning diet on the age at puberty onset, first service and age at conception. Since the age at first service and age at conception are in part affected by management practices such as heat detection, survival analysis was used to investigate the age at which animals reached pre-determined height (125 cm withers height) and weight (380 kg) targets after which they were eligible for insemination. Kaplan Meier survival curves were plotted for Group A and R separately. Multivariable Cox proportional hazard models were fitted for all outcomes to assess the hazard ratio for potential explanatory variables. Variable selection for final models was by backwards stepwise removal taking a *P* value < 0.200 (log likelihood test) for retention of a variable. The following explanatory variables were offered to all models: Birth weight, dam parity (primiparous *v* multiparous), plasma TP and occurrence of diarrhoea and/or pneumonia during the first 12 weeks. Dietary group was forced into all models. Proportional hazard assumptions for Cox’s regression were checked using Schoenfeld residuals and were accepted if P > 0.050.

## Results

The mean birth weight of heifer calves (*n* = 100) was 41.78 kg (95% CI 40.66–42.90). Birth weight was positively associated with dam parity (primiparous: *n* = 43, mean 38.4 kg, 95% CI 37.1–39.8; multiparous: *n* = 57, mean 44.3 kg, 95% CI 42.9–45.7, P < 0.001).

Time from birth to first colostrum ingestion was similar for calves in both dietary groups (Group R: 3.29 hours, range 0.50–11 hours, Group A: 3.27 hours, range 0.25–9.50, P = 0.974). The specific gravity of *peri-partum* colostrum was comparable between dietary groups (23.5%, 95% CI 22.5–24.5) and was not influenced by dam parity. Calves born to primiparous dams consumed less colostrum in their first meal (primiparous: 2.96 L, 95% CI 2.77–3.15 L; multiparous: 3.26 L, 95% CI 3.11–3.40 L, P = 0.006). However, when data were normalised for calf body weight, initial colostrum intakes were similar for all calves (primiparous dams: 0.077 litres/kg BM; multiparous dams: 0.075 litres/kg BM, P = 0.337). Mean PTP (sd) concentration was 68.9 g/L (0.81) with no dietary group (P = 0.76) or dam parity associated (P = 0.91) differences. Taking a PTP cut-off of 56.0 g/L as indicative of adequate passive transfer, 5 (5.0%) calves were classified as having FPT. There were no associations between occurrence of FPT and calf group (P = 1.000) or dam parity (P = 0.650).

**Fig 1:**
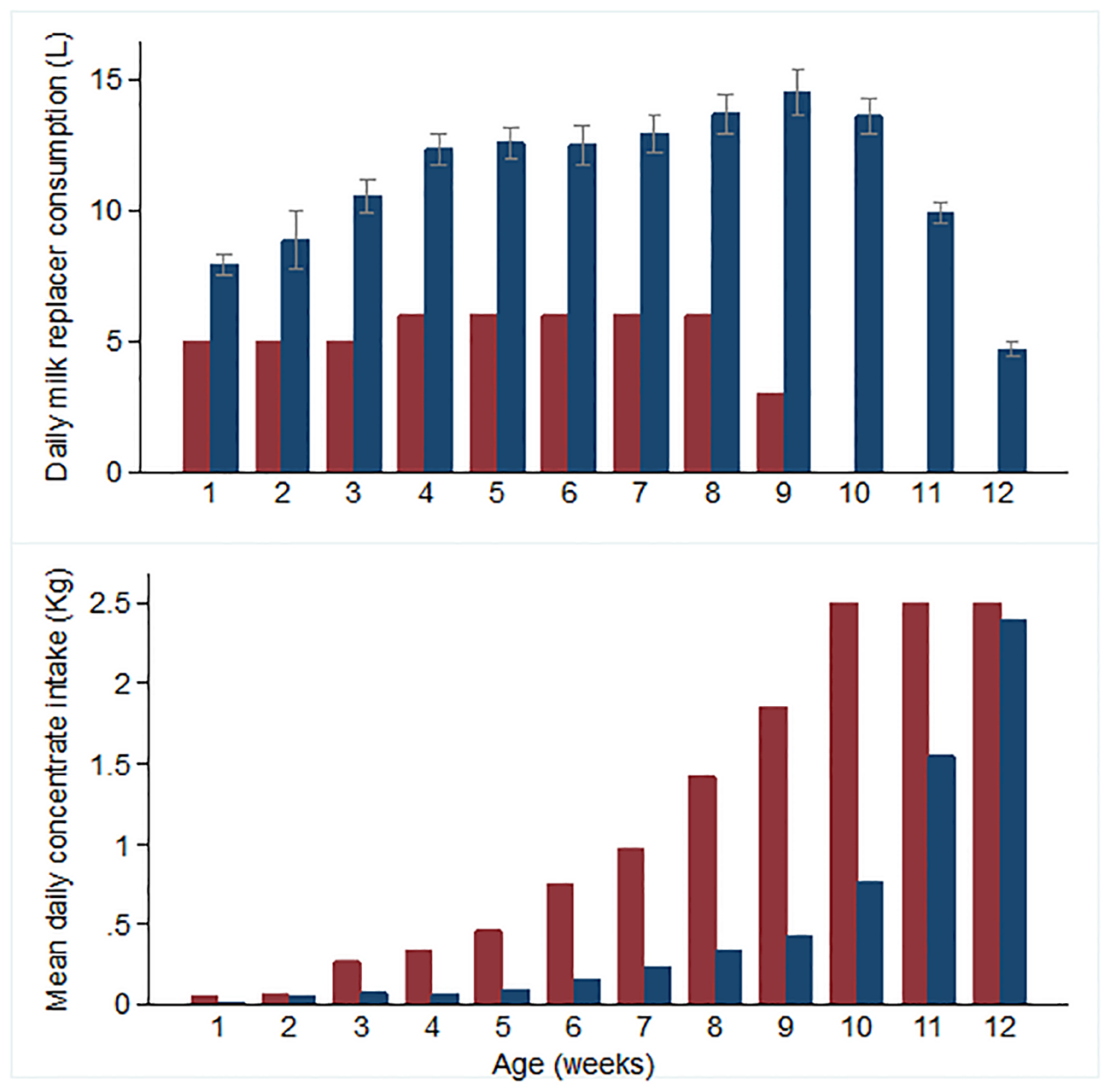
Milk replacer and calf concentrate consumption for Group A and Group R calves. a) Mean (95%CI) daily MR consumption for heifer calves in Group A (blue), and MR allowance for Group R animals (red) and b) mean weekly concentrate intakes (kg) for a subset of calves in Group A (blue, *n* = 9) and Group R (red, *n* = 7) from birth until 12 weeks of age.

All calves consumed all transitional milk offered during the first 4 days of life. Group A calves consumed considerably more MR (mean 914 litres, 95% CI 873–947) than Group R animals (315 litres) over the entire pre-weaning period (Fig 1a). Voluntary daily MR intakes in Group A calves increased rapidly to reach 7.6 L/day (95% CI 6.5–8.7) by day 5 and increased linearly to reach 13.3 L (95% CI 12.4–14.2) by day 26 before the rate of increase slowed to peak at 15.3 litres per day (95% CI 14.2–16.4) near the onset of gradual weaning on day 64. The maximum daily MR intake recorded for any calf was 25.5 litres. In Group A, MR intakes declined at a pre-programmed rate of 0.7 L daily (Fig 1a) over a 21 day period, whilst Group R calves had a 50% reduction in MR supply for one week prior to weaning at 63 days of age. When data were corrected for body weight and ME, the maximum mean energy provision from MR at 3 weeks of age for calves in Group A was 0.54 MJ/kg body weight/day compared to 0.34 MJ/ kg body weight/day in Group R.

Concentrate feed intakes were negligible (< 50g) for calves in both dietary groups from birth to 3 weeks. After this time, voluntary intakes of concentrate feed for Group R calves gradually increased to approximately 1.0 kg daily by 7 weeks of age. Conversely, concentrate intakes were relatively less for Group A calves and had only attained < 0.5 kg/head daily by the onset of weaning at week 8 (Fig 1b). However, mean intakes were similar in both groups of calves at 12 weeks of age i.e at completion of weaning.

There was a high incidence of disease in both groups of calves. In total, 80 (80%) calves suffered from at least one incident of disease during the period from birth to 12 weeks. Group A calves had a greater risk of disease than group R calves (diarrhoea: OR 3.86, 95% CI 1.67 to 8.9; pneumonia: OR 5.80, 95% CI 2.33 to 14.44) [28]. However, no animals died during the pre-weaning period. Of the 100 heifer calves which entered the study, 98 animals remained within the cohort at the end of the study period (68.9 weeks, 95% CI 66.6–71.3). Two animals, one from each dietary group, died post-weaning prior to reaching eligibility for first service (accidental death), these animals were excluded from further analyses.

Overall, pre-weaning growth rates (kg/day) (Table 2) were significantly higher in Group A calves (0.89 kg/day, 95% CI 0.86–0.93: measured over 12 weeks) compared to Group R (0.57 kg/day, 95% CI 0.54–0.6: measured over 9 weeks) such that at 12 weeks of age, when weaning was complete in both groups, there was a significant difference (P <0.0001) in mean body weight between the 2 groups (Group R: 103.88 kg, 95% CI 100.55–107.21, Group A: 116.80 kg 95%CI 113.45–120.15). However, mean daily growth rates showed temporal variation between the 2 groups during the pre-weaning period. The most marked differences were observed during the first three weeks of life (Group A 0.72 kg/day, 95% CI 0.61–0.82, Group R 0.17 kg/day, 95% CI 0.08–0.26, P < 0.001). Conversely, between 9 and 12 weeks of age when Group A were undergoing gradual weaning, growth rates were greater in Group R (Group R: 1.04 kg/day, 95% CI 0.97–1.11, Group A: 0.84 kg/day, 95% CI 0.74–0.93, P < 0.001).

**Table 2:** Mean daily live weight gains from birth until 12 weeks. Daily live weight gains for heifer calves in both Group A and R week split into 4 periods throughout the first 12 weeks of life.

Age (weeks)	Mean daily live weight gain (kg) 95% CI		P Value
	Group R (*n* = 50)	Group A (*n* = 50)	
0.00–2.99	0.17 (0.08–0.26)	0.72 (0.61–0.82)	<0.001
3.00–5.99	0.74 (0.68–0.81)	0.91 (0.83–0.99)	0.001
6.00–8.99	0.92 (0.86–0.98)	1.04 (0.94–1.13)	0.019
9.00–11.99	1.04 (0.97–1.11)	0.84 (0.74–0.93)	<0.001

**Fig 2:**
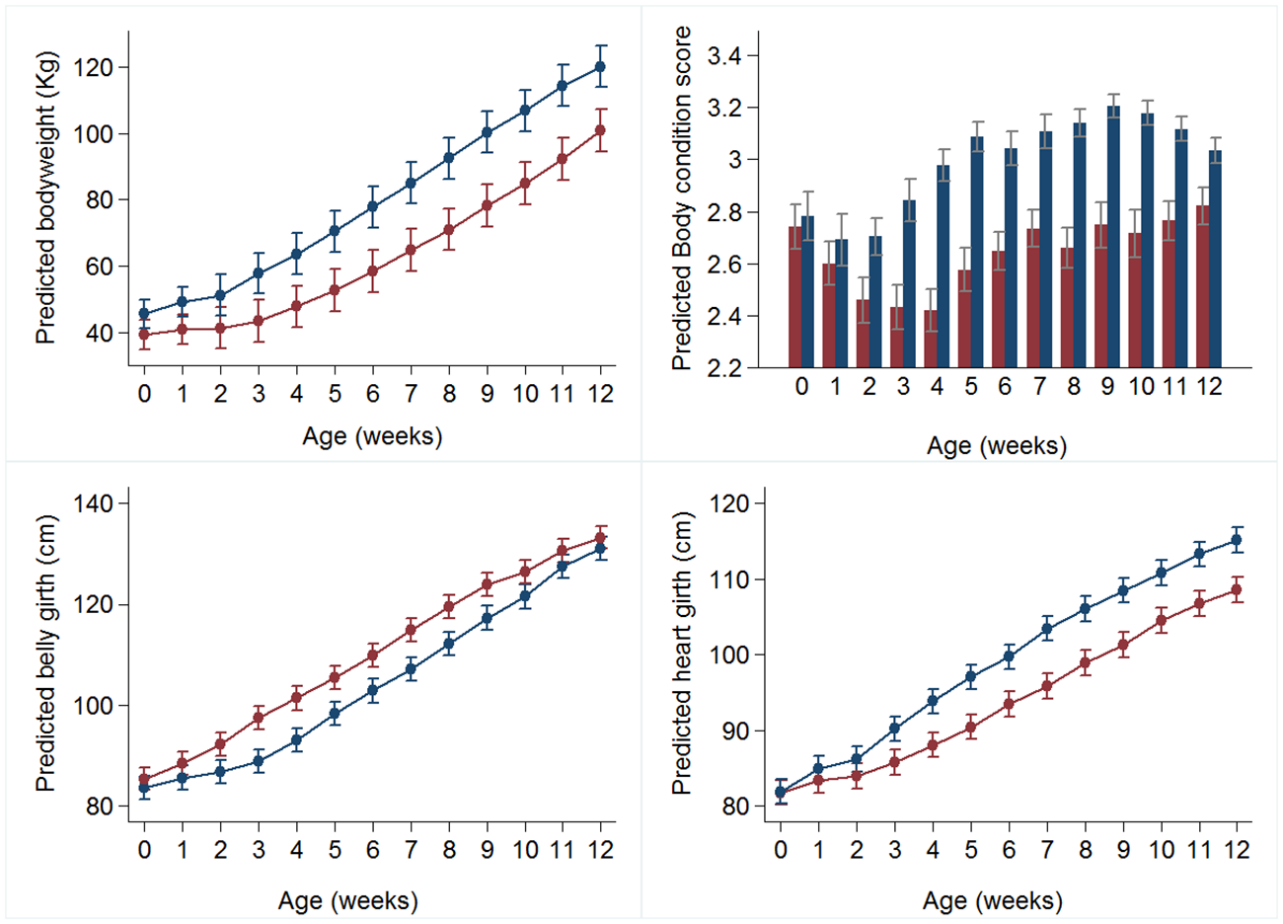
Predicted marginal means (95% CI) for morphometric measures. a) body weight (kg), b) BCS c) belly girth (cm), and d) heart girth (cm) for calves in Group A (blue) and R (red) from birth until 12 weeks of age. Full models are presented in S1 supporting information.

Multivariable modelling (Tables 3 and 4, Fig 2) suggested the following explanatory variables were associated with body weight change over the first 12 weeks of life: pre-weaning dietary group (*ad libitum* or restricted MR) with an interaction with age in weeks, dam parity, plasma TP, the presence of diarrhoea in the first 12 weeks and presence of pneumonia in the first 12 weeks. A plot of the model-derived predicted marginal means for body weight (Fig 2a) indicated that the impact of dietary group was most marked during the first 3 weeks of life when increase in body weight was minimal for Group R animals. Beyond this time, rate of change in body weight were broadly similar between pre-weaning dietary groups. Early constraints on growth therefore resulted in a right shift of the growth curve for Group R.

**Table 3:** Multivariable regression model for the association between body weight and pre-weaning dietary group. Multivariable regression model for body weight and its association with dietary group from 0 to 108 weeks of life, including potential confounders. Calf was included as a random effect. The residual variance attributed to individual calf ID was 34.3% (95% CI 28.0–41.4). Coefficients for time (in weeks) and interaction terms are omitted for clarity.

Outcome variable: Body weight	Coefficient	95% Confidence Intervals	P value
Dietary group (*ad libitum* or restricted MR)	5.740	-0.842–12.322	0.087
Dam parity (multiparous *vs* primiparous)	7.692	2.448–12.936	0.004
Plasma TP	3.626	0.391–6.860	0.028
Pneumonia (first 12 weeks)	-8.431	-14.616–-2.246	0.008
Diarrhoea (first 12 weeks)	-4.521	-10.184–1.142	0.118
Constant	15.958	-6.610–38.526	0.166

**Table 4:** Random-effects parameters for multivariable regression model for the association between body weight and pre-weaning dietary group.

Random-effects Parameters (variance)	Estimate	95% Confidence Intervals
calf:	157.160	117.024–211.062
Residual	300.417	287.161–314.286

There were marked dietary group differences in BCS change during the first four weeks of life (Fig 2a), with Group A calves gaining BCS at a rate 0.0053 (sd 0.0190) units per day compared to Group R calves who lost BCS at a rate 0.0149 (sd 0.0207) units per day (P <0.001) during this period. From 3 weeks onwards, BCS increased for both dietary groups.

**Fig 3:**
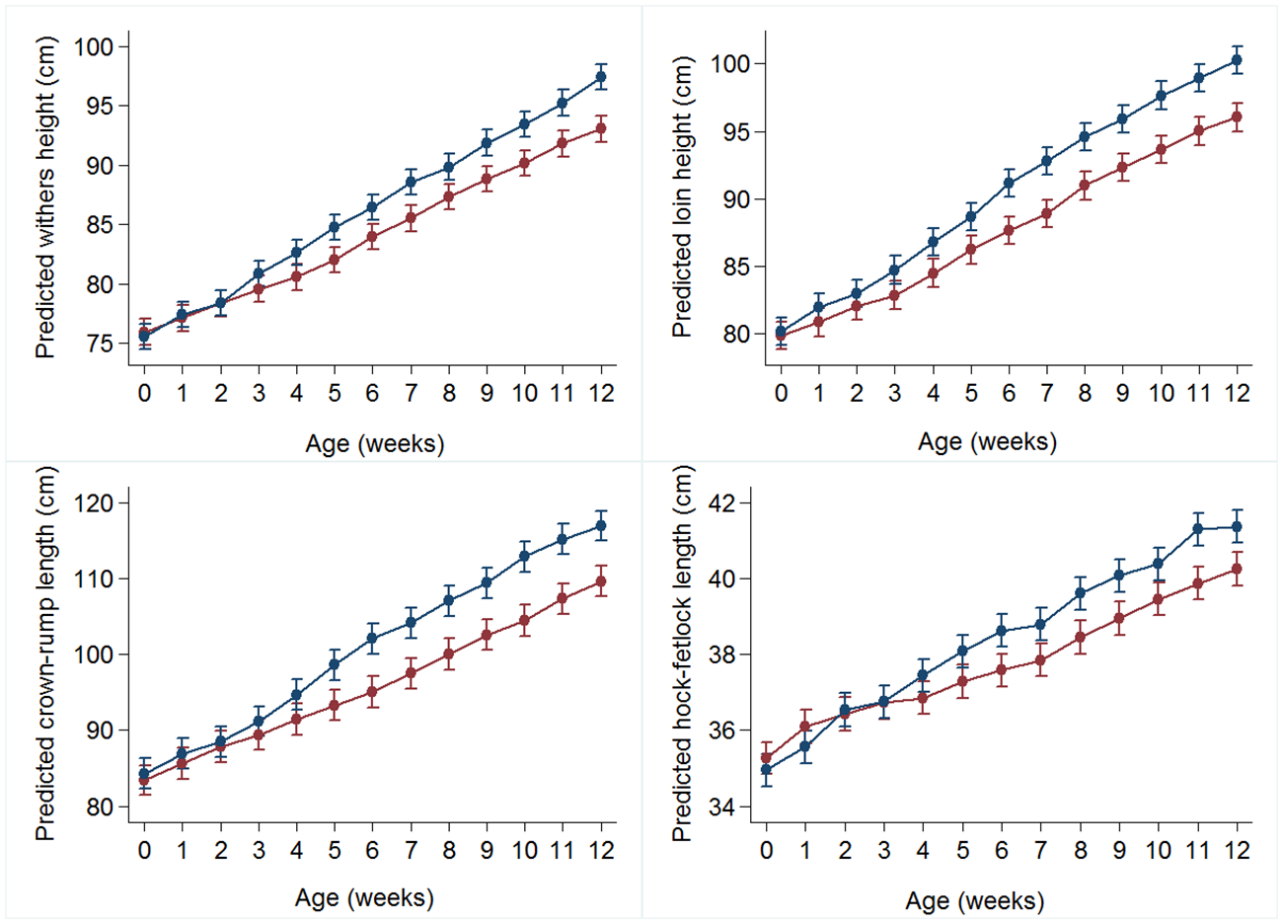
Predicted marginal means (95% CI) for skeletal morphometric measures. a) withers height (cm), b) loin height (cm) c) crown-rump length (cm), and d) hock-fetlock length (cm) for calves in Group A (blue) and R (red) from birth until 12 weeks of age. Full models are presented in S1 supporting information.

With the exception of belly girth, all other morphometric measures (withers and loin height, heart girth, CRL and HFL) had broadly similar dietary group differences in rates of change over time during the first 12 weeks of life, although group differences in predicted withers height and other skeletal measures were only apparent after 3 weeks of age (Fig 3).

Mean average daily weight gain from 12 to 60 weeks (Group R, *n* = 49; Group A, *n* = 49) was 0.837 kg daily (95% CI, 0.815–0.860) with no dietary group differences (P = 0.771). For all other morphometric measures including body weight, Group A calves had higher recorded values than Group R calves from 12–60 weeks, but these differences were not statistically significant at every time point. There was a trend for pre-weaning diet-associated differences to decrease over time, such that by the end of the study there were no differences attributed to pre-weaning dietary group in any of the morphometric measures with the exception of body weight. Although mean BCS remained higher in Group A than Group R animals throughout the study period, there was a trend for BCS to increase in both dietary groups from approximately 36 weeks of age onwards (S2 supporting information).

**Fig 4:**
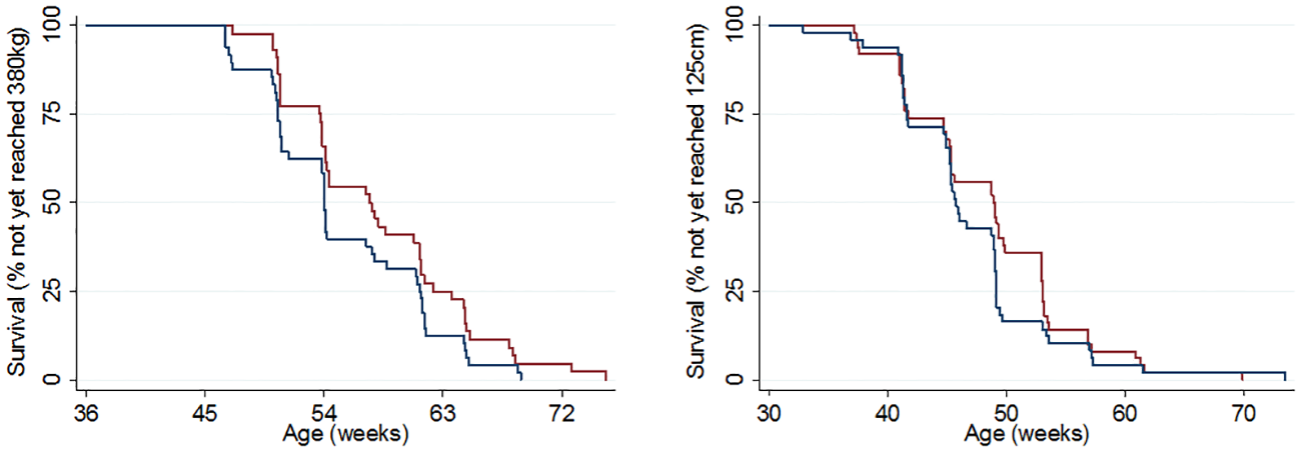
Kaplan- Meier survival curves for morphometric measures relating to first service parameters. Proportion of heifers not yet reaching a) 380 kg and b) 125cm withers height in Group A (blue line) and Group R (red line).

**Fig 5:**
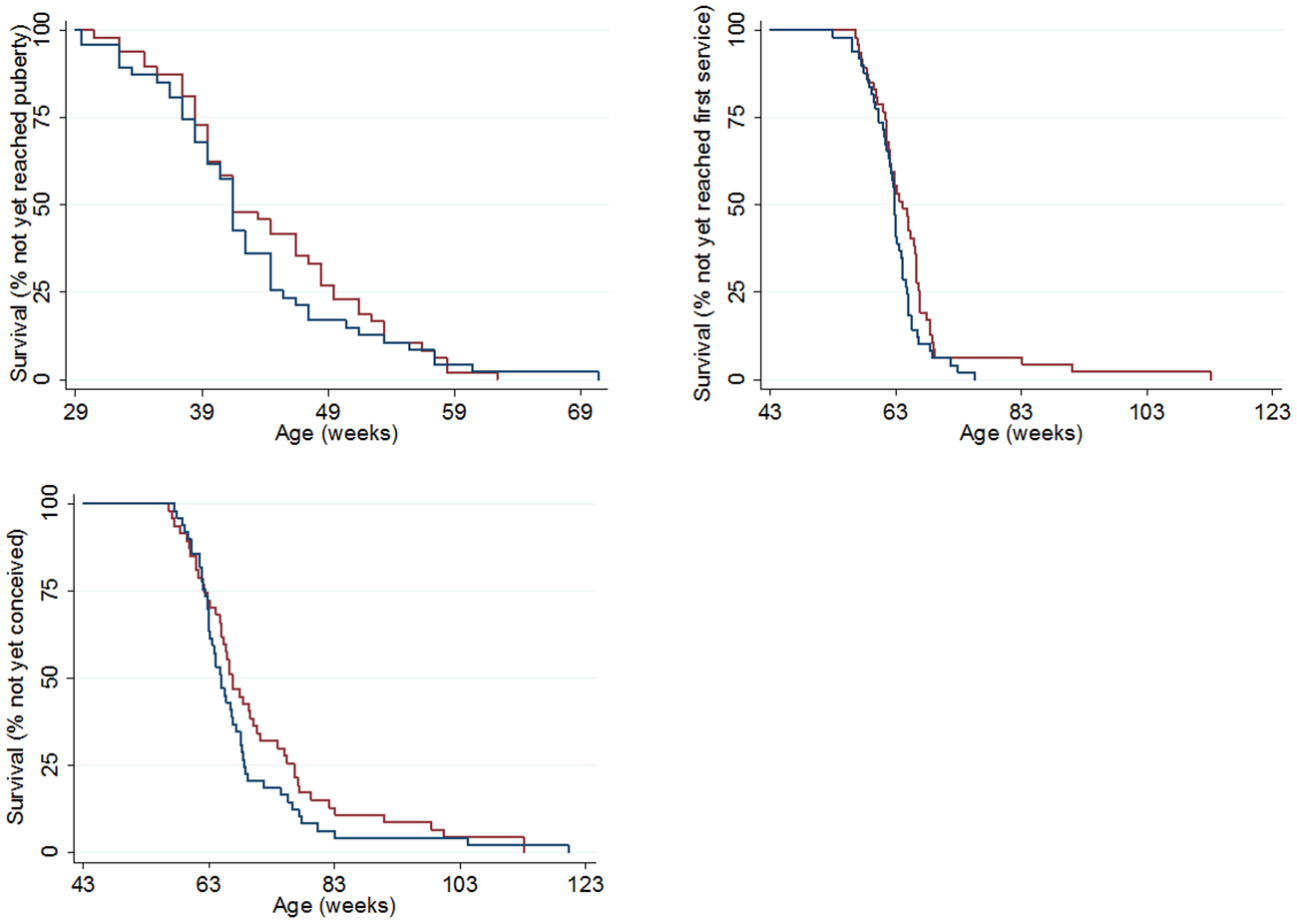
Kaplan- Meier survival curves for measured KPI’s. Proportion of heifers not yet reaching a) puberty, b) first service and c) conception in Group A (blue line) and Group R (red line).

The mean age at which animals reached 380 kg (pre-defined as the minimum weight for insemination) was 57.4 (95% CI 55.4–59.4) weeks for Group A calves and 60.4 (95% CI 58.2–62.7) weeks for Group R calves (P = 0.043) (Fig 4). However, the mean age at which animals reached 125 cm height at the withers (pre-defined as the minimum withers height for insemination) was 46.5 (95% CI 45.1–47.8) weeks with no dietary group difference (P = 0.230) (Fig 4). The mean age at the onset of puberty was 41.6 (95% CI 39.2–44.1) weeks for Group A calves and 43.8 (95% CI 41.6–46.0) weeks for Group R calves (P = 0.167) and was not affected by disease (diarrhoea or pneumonia) during the pre-weaning period (P = 0.469). The mean age at first service was 62.6 weeks (95% CI 61.4–63.8) for Group A and 65.3 (95% CI 62.6–68.0) weeks for Group R (P = 0.068) and the age at conception was 67.7 (95% CI 64.5–70.8) weeks for Group A and 70.1 (95% CI 66.6–73.6) weeks for Group R (P = 0.305) (Fig 5). Based on a 285 day gestation period, predicted calving ages for heifers from Group A was 25.3 months (95% CI 24.6–26.0), and Group R animals was 25.8 months (95% CI 25.0–26.7). Neither age at first service or conception were affected by disease during the pre-weaning period. Forty-nine animals in Group A and 46 animals in Group R conceived.

The mean number of services required to achieve conception was similar between dietary groups (2.02; 95% CI 1.66–2.38, P = 0.998). Fifty seven percent of group A and 51% of Group R animals became pregnant after the first service (P = 0.550). There were no dietary group differences in body weight, withers height or BCS at the onset of puberty, first service and conception (P > 0.050) (Table 5).

**Table 5:** Key Performance Indicators at key reproductive stages for heifers in both pre-weaning groups. Mean (95% CI) age, body weight, withers height and body condition score for all heifers at 3 key events in the study; the onset of puberty, first service and conception.

	*Ad libitum* fed MR	Restricted fed MR	P value
**Puberty**			
Age (weeks)	41.6 (39.2–44.1)	43.8 (41.6–46.0)	0.167
Body weight (kg)	292.3 (279.4–305.1)	287.6 (276.5–298.6)	0.289
Withers height	122.4 (120.5–124.3)	123.7 (122.2–125.2)	0.145
BCS	2.92 (2.84–3.00)	2.90 (2.82–2.98)	0.371
**First service**			
Age (weeks)	62.6 (61.4–63.8)	65.3 (62.6–68.0)	0.068
Body weight (kg)	418.5 (409.3–427.7)	409.6 (395.7–423.5)	0.141
Withers height	133.4 (132.3–134.5)	133.7 (132.5–134.9)	0.349
BCS	3.08 (3.01–3.15)	3.06 (2.98–3.13)	0.290
**Conception**			
Age (weeks)	67.7 (64.5–70.8)	70.1 (66.6–73.6)	0.305
Body weight (kg)	443.0 (426.9–459.1)	443.0 (422.4–463.6)	0.500
Withers height	135.0 (133.8–136.2)	135.8 (134.3–137.4)	0.196
BCS	3.14 (3.04–3.23)	3.06 (2.98–3.14)	0.118

Multivariable Cox regression (Table 6) suggested that in the case of time to achievement of 380 kg target weight there was a significant association with dietary group (Hazard ratio 1.7 95% CI 1.08–2.67, P = 0.021) but this was not the case with withers height (P = 0.330). In the case of time to achievement of puberty there were statistically weak associations with dietary group (Hazard ratio 1.14, 95% CI 0.88–2.26, P = 0.157) and occurrence of pneumonia during the pre-weaning period (Hazard ratio 0.70 95% CI 0.43–1.14, P = 0.147). However, time to first service was associated with dietary group (Hazard ratio 1.47, 95% CI 0.97–2.24, P = 0.072), birth weight (Hazard ratio 1.08 95% CI 1.03–1.14 P = 0.001), plasma total protein concentration measured at 48 hours of age (Hazard ratio 1.43 95% CI 1.09–1.87 P = 0.009). Time to conception was weakly associated with dietary group (Hazard ratio 1.39, 95% CI 0.93–2.1, P = 0.109) and significantly associated with birth weight (Hazard ratio 1.09, 95% CI 1.04–1.15, P < 0.001) (Table 6). In the case of all outcomes apart from time to puberty, there was a significant and consistent negative impact of having a multiparous dam with hazard ratios ranging from 0.44–0.67.

**Table 6:** Cox regression models for time to achieving pre-determined morphometric targets, puberty, first service and conception.

	Hazard ratio	95% Confidence Interval	P value
**Time to withers height of 125 cm**			
Dietary group (*ad libitum* or restricted MR)	1.23	0.82–1.84	0.33
Birth weight (kg)	1.09	1.04–1.14	< 0.001
Dam Parity (multiparous *vs* primiparous)	0.44	0.27–0.74	0.002
**Time to body weight of 380 kg**			
Dietary group (*ad libitum* or restricted MR)	1.70	1.08–2.67	0.021
Birth weight (kg)	1.09	1.04–1.15	0.001
Dam Parity (multiparous *vs* primiparous)	0.51	0.29–0.91	0.022
Plasma TP	1.25	0.95–1.63	0.105
Pneumonia during pre-weaning period	0.78	0.55–1.11	0.17
**Time to puberty**			
Dietary group (*ad libitum* or restricted MR)	1.41	0.88–2.26	0.157
Pneumonia during pre-weaning period	0.70	0.43–1.14	0.147
**Time to First Service**			
Dietary group (*ad libitum* or restricted MR)	1.47	0.97–2.24	0.072
Birth weight (kg)	1.08	1.03–1.14	0.001
Dam Parity (multiparous *vs* primiparous)	0.58	0.34–0.99	0.045
Plasma TP	1.43	1.09–1.87	0.009
**Time to conception**			
Dietary group (*ad libitum* or restricted MR)	1.39	0.93–2.1	0.109
Birth weight (kg)	1.09	1.04–1.15	< 0.001
Dam Parity (multiparous *vs* primiparous)	0.67	0.41–1.1	0.114

## Discussion

This study was designed to investigate the impact of *ad libitum* milk replacer (MR) feeding and group housing from birth (*n* ≤ 6/group) on the growth and performance of Holstein dairy calves under commercial conditions. For comparison, a control group of calves fed restricted amounts of MR housed from birth until 3 weeks and thereafter grouped (*n* ≤6) and fed restricted MR according to current U.K. practices was also studied. The decision to base the intervention on *ad libitum* MR feeding rather than increased volumes was made on the basis that it was important to understand the growth potential of Holstein calves with access to unlimited MR. It was not the intention of the study to provide a novel “off the shelf” feeding strategy for adoption by the farming industry.

The present study provides valuable information regarding impact of feeding and housing systems on growth and future reproductive performance, despite there being implicit study limitations which must be acknowledged. Firstly, calves were not randomly allocated to intervention arm. This was dictated by the farm size and calving rate, coupled with the requirement to produce groups of up to six calves with no more than 14 days’ age difference between them. Secondly, due to the standard farm policy for housing calves individually during the first 3 weeks of life, there is implicit confounding of feeding system by housing and *vice versa* thus not allowing differences to be attributed absolutely to either feeding system or housing system. Another shortcoming of the study is the relatively small sample size, dictated by the study farm size. This reduces the statistical power and ability to detect differences at the P < 0.05 level. In fact, post-hoc power calculations suggest that whilst the sample size (*n* = 100) is adequate (> 80% power) to detect differences in body weight and growth rates during the study period, it is insufficient to detect significant differences in achieving specific reproductive or growth targets; having a power of 53% to detect a hazard ratio of 1.5, with only a 17% power to detect a 10% difference in e.g. conception rates. For this reason we present actual P values allowing the reader to judge the validity of conclusions reached.

There is an increasing body of evidence in support of increased milk or MR feeding of dairy calves during early life with the majority reporting benefits in terms of increased daily live weight gain (DLWG) during the pre-weaning period [14,29–31]. Current targets for DLWG for the entire pre-weaning phase are 0.8–0.9 kg/day [32]. In the current study, all calves were weighed and measured weekly affording the opportunity to investigate in depth morphometric changes during the pre-weaning phase. Overall, mean pre-weaning growth rates were more than 1.5 times greater in Group A calves (0.89 kg/day, 95% CI 0.86–0.93: measured over 12 weeks) compared to Group R (0.57 kg/day, 95% CI 0.54–6.00: measured over 9 weeks), such that at 12 weeks of age, when weaning was complete for all calves, Group A calves were 13 kg (12%) heavier on average compared to their Group R counterparts. The most dramatic differences in growth rates were observed during the first 3 weeks of life when DLWG was 0.72 kg (95% CI 0.61–0.82) per day in the *ad libitum* MR fed, group housed calves compared to 0.17 kg (95% CI 0.08–0.26) per day in the restricted MR fed, individually penned calves, a four-fold difference, during this period. Similar findings have been reported previously [14]. This minimal growth in the restricted MR fed calves was accompanied by a considerable loss in BCS of nearly 0.5 BCS points over the first 4 weeks, comparable to that of an adult lactating cow during the first 8 to 10 weeks *post-partum*. In contrast, a consistent increase in BCS from birth was recorded in the *ad libitum* MR fed animals. In addition to the differences in body weight gain, other morphometric measures of growth differed between dietary groups during the pre-weaning period. In the case of skeletal measures namely withers and loin height, heart girth, crown to rump length and hock-fetlock length, there was no dietary group difference in the first 3 weeks of life, in contrast to body weight. However from 4–6 weeks of age *ad libitum* fed calves demonstrated significantly increased skeletal growth compared to their restricted fed counterparts. This observation, together with the BCS changes observed in early life in the restricted fed calves, would suggest that skeletal growth is prioritised at the expense of soft tissue growth during the first 3 weeks of life.

After three weeks of age solid food intake increased in the restricted MR fed calves coincident with an increased growth rate comparable to that of the *ad libitum* MR fed calves. However their body weights remained lower with no compensatory growth observed throughout the pre-weaning period. The ability for ‘catch-up’ or ‘compensatory’ growth following a period of limited nutrient availability is well documented for various mammalian species [33,34]. Abdalla *et al* [35] demonstrated that compensatory growth occurred in Holstein calves, aged at least 8 weeks at recruitment, that underwent a period of restriction of protein and energy intake sufficient to reduce daily live weight gain by 50% for between 112 and 154 days. In the present study, nutritional restriction was applied to the restricted fed calves from birth, supporting the hypothesis that compensatory growth cannot occur if dietary restriction is imposed during very early life [15,36].

As previously mentioned, the effect of dietary intake during the first 3 weeks of life is confounded by housing as calves fed restricted volumes of MR were single penned in contrast to the *ad libitum* fed calves which were group housed from birth. It has been clearly shown that growth, solid feed intake and social development of calves are all improved by group housing calves compared to individual penning [37]. Thus it could be concluded that group housing in combination with unrestricted MR feeding affords both welfare and financial benefit.

Rates of both diarrhoea and pneumonia were significantly higher in the Group A compared to Group R calves. In the case of diarrhoea, all cases occurred during the first 3 weeks of life when Group R calves were individually penned compared to their Group A counterparts who were group penned. Diarrhoeic calves were not removed from group pens, so it is likely that the higher rates observed were a reflection, in part, of a greater pathogen transmission potential. However it is recognised that feeding increased amounts of milk or MR will result in greater volumes of looser faeces. Thus it is also possible that since diagnosis of diarrhoea was solely on the basis of a faecal score > 2 that some calves in Group A were mis-classified as suffering from diarrhoea when in fact they were not, further adding to the number of cases observed in this group. In fact, there is little evidence regarding any deleterious impact *per se* of a high plane of nutrition on risk of diarrhoea [14,29], whilst there is evidence that increased plane of nutrition can mitigate the severity of experimentally induced diarrhoea associated with *Cryptosporidium parvum* infection [38]. However the increased risks of disease transmission in group housed animals are well recognised [39]. In the case of pneumonia, all episodes were observed in calves over 3 weeks of age. All calves were housed in a common air space which suffered from high humidity and wide temperature fluctuations, coupled with poor drainage; all of which are well recognised risks for pneumonia. We hypothesise that the increased pneumonia risk in the *ad libitum* milk fed calves was likely associated primarily with the sharing of a common teat facilitating transmission of respiratory viruses via saliva and nasal secretions [40]. A further factor could be the increased volumes of urine produced associated with increased fluid intakes leading to increased bed wetness. Disease risk in the present study is the topic of a previous publication to which the reader is referred for further details [28]. The published work on the impact of group housing on disease risk is contradictory with some studies [41–43] showing increased disease risk associated with group housing, whilst others have reported improved health in group housed calves [44,45]. Increasing group size [46] and continual introduction of calves into the group versus an all in—all out stable grouping system [47] are both associated with increased disease risk. In the present study, group size was equal to or less than 6 with no more than 14 days age difference and all groups were stable, thus mitigating these latter risks.

Restricted feeding of milk or MR has traditionally been justified on the basis of promoting early rumen development associated with consumption of concentrate feed at an early age [48]. New born calves are mono-gastric with undeveloped rumens thus are incapable of utilising solid foods until the establishment of a rumen microflora and rumen villous growth has occurred. Concentrate intakes during the first 3 weeks of life were minimal in calves of both dietary groups (Fig 1) corresponding to this so-called pre-ruminant phase [21], during which the calf is dependent on MR for almost all its nutritional requirements. This inability of the young calf (< 3 weeks old) to utilise solid feeds will accentuate the energy deficits associated with restricted MR feeding and contribute to the loss in BCS observed in these calves. This would imply that under-feeding of MR at this stage is not compatible with the concept of a “suitable diet” as stipulated by their five needs [49,50].

A concern raised with regards to feeding increased volumes of milk or MR is that calves will have poor rumen development at the time of weaning due to insufficient consumption of concentrate feed [51–53]. In the present study, a three week step-down weaning strategy was adopted for the *ad libitum* MR fed calves in order to minimise this risk. Concentrate intakes in *ad libitum* MR fed animals was relatively low prior to the onset of weaning, but increased rapidly to that of restricted MR fed calves by the end of the pre-weaned period with no apparent adverse effects on health.

Plasma total protein concentration at 48 hours of age had a significant positive impact on growth up to the time of conception, although this was not apparent during the first 12 weeks of life. The impact of PTP on long term growth was such that it remained an explanatory variable in the Cox regression model for time to first service. These findings suggest that colostrum intake impacts not only on the immune status of the calf *via* transfer of immunoglobulins, but has other, at present poorly described, roles affecting future growth. Similar findings have been shown by other groups and it is likely that these findings illustrate a further mechanism by which early life colostrum intake has a long term impact on animal lifetime production [15,54].

Whilst the intervention was applied during the pre-weaning period, data collection continued until conception, as confirmed by ultrasound examination. In terms of body weight, there was no clear evidence of any catch-up growth in the restricted MR fed group as demonstrated by the earlier age at which Group A animals achieved a target weight of 380 kg. Interestingly, differences in other morphometric measures between the 2 dietary groups disappeared over time such that there were no group differences in age of achievement of the 125 cm withers height target. This suggests a degree of catch up growth occured in respect of skeletal measures. It is unclear why catch-up growth occurred with respect to morphometric measures (which included measures of skeletal growth) but not for body weight.

While the feeding regime for study animals during the pre-weaning and early post-weaning phases were highly controlled, this was not the case from 5 months of age onwards when all heifers were fed ‘left-over’ Total Mixed Ration (TMR) from the adult lactating and dry cow diets which will have likely varied considerably in composition especially with respect to dietary starch levels. Due to this variablility it is not possible to accurately state what the likely energy density or protein content of the diet was: however the lactating cow diet fed had an approximate energy density of 12 MJ ME/ kg DM which is considerably higher than required for growing dairy heifers at this stage of life, which is in the region of 9.5–10.5 MJ/kg DM [55]. This may have contributed to the increase in BCS observed in both groups from 4 months of age onwards.

One major Key Performance Indicator (KPI) associated with dairy heifer rearing is age at first calving (AFC). Most published studies suggest an optimal AFC of 23 to 24 months [56], although a recent study based on over 400,000 animals in the UK suggest that an AFC of 22–23 months may be associated with increased lifetime productivity [57]. In the present study, predicted AFC was sub-optimal in both groups of animals at 25.3 months for Group A compared to 25.9 months for Group R. However irrespective of dietary group mean weight at first service was 414 kg i.e. 34 kg over the 380 kg target. Assuming an average daily weight gain of 0.85 kg per day—this would imply that heifers were first served on average 40 days after reaching their target weight. This would suggest that improved heat detection would dramatically reduce AFC by an average of 40 days. Prior to commencement of the current study, a consensus was reached that there were 2 targets to be met prior to first service, namely attainment of a body weight of 380 kg and a withers height of 125cm. These targets were intended to be employed on the farm for the duration of the study. However, farm staff failed to utilise weight and height data collected by the researcher for decision making regarding breeding. This likely had a deleterious impact on the achievement of optimal age at conception. When retrospectively evaluating final morphometric measures from study heifers it was interesting to find that only 40 out of the 100 heifers reached the 2 targets simultaneously. Generally, the height target was reached earlier than the body weight measure; survival curves presented (Fig 4) suggest there was up to 4 weeks difference between achievement of the 2 targets. This raises the question: which is the best KPI measure for timing of first service—body weight or withers height?

There were statistically non-significant trends for *ad libitum* MR fed animals to reach all reproductive KPIs (age at puberty, age at first service and age at conception) earlier than restricted MR fed animals. The *ad libitum* MR fed heifers achieved targets approximately 2–3 weeks earlier than their restricted MR fed counterparts, suggesting significant financial benefits could accrue from the increased feeding regime. Although not statistically significant, *ad libitum* MR fed heifers had a higher conception rate (57%) than restricted MR fed heifers (51%). This lack of statistical signifcance may be a reflection of the relatively small sample size (*n* = 50 per group). Although the *ad libitum* MR fed heifers reached the pre-defined target weight of 380kg earlier than their restricted fed counterparts, the target AFC of 24 months was not met by either group as discussed above due to delay in serving heifers on achievement of their target weight. It is imperative that if the benefits of improved early life growth are to be realised, farm management practices must be optimised to ensure heifers are served as early as possible after becoming eligible. The target should be that all heifers are served within 21 days of achieving target weight or height. This requires both regular monitoring of height and/or body weight of heifers and active heat detection. A recent trend has been the development of hormonal interventions to allow fixed time insemination thereby eliminating the need for oestrus detection [58].

Multivariable cox regression was performed to investigate factors associated with time to achievement of the pre-determined morphometric measures and reproductive parameters. Whilst caution must be taken in interpretation of these models due to the small sample size, it is interesting to note that for all outcomes, except “time to puberty” there was a negative association (hazard ratio less than 1) with the binary variable “parity” having adjusted for birthweight. This suggested that the hazard for heifers born from multiparous dams was 20–40% less than for those from primiparous dams for achievement of these targets. This finding is in contrast to the impact of dam parity on weight over the study period as shown by the positive coefficient (7.69 Table 3). However this is likely to be a proxy for the heavier birthweight of calves born from multiparous dams compared to those from primiparous dams (44.3 vs 38.4 kg in the present study). This observation is in agreement with those of Swali & Wathes [59] who also found time to conception was significantly less in heifers born to primiparous dams. Two hypotheses can be generated regarding this finding; firstly it is associated with *in utero* nutrition either via a direct effect on ovarian development [60] or indirectly via unknown epigenetic mechanisms. Alternatively it could be associated with differences between colostrum derived from primiparous compared to multiparous dams. A recent study [61] highlighted the reduced microbial richness in multiparous dam colostrum compared to primiparous colostrum, suggesting this may be a consequence of routine intra-mammary antimicrobial treatment of lactating cows at the end of lactation (dry cow therapy). Furthermore it is recognised that the colostrum microbiome will contribute to the calf intestinal microbiome, the composition of which can impact on gut health and early life growth [62].

In the current study, *ad libitum* MR fed heifers reached conception and therefore predicted first calving 2.3 weeks earlier than restricted MR fed heifers. Based on current estimates of the financial losses associated with a delayed AFC (£2.87/day [32]), this equates to an accrued financial benefit of £46.21 per *ad libitum* fed heifer. However the approximate additional cost of *ad libitum* feeding for 12 weeks compared to restricted MR feeding and weaning at 9 weeks was £84 (£187 *versus* £103), suggesting that in this study, at least, the financial benefits of a reduced AFC did not justify the increased rearing costs associated with *ad libitum* MR feeding and weaning at 12 weeks of age. Thus, this study demonstrated clear benefits of *ad libitum* feeding on growth and by extension calf welfare during the first 3 weeks of life but failed to demonstrate a clear financial benefit. However since the study ended when animals achieved pregnancy, no data regarding future health or production is available. There is an increasing body of evidence reporting reported the benefits of increased liquid milk feeding for at least the first 5 weeks of life on lifetime performance [15,20,63].

With this in mind, future studies should investigate the impact of shorter *ad libitum* MR feeding periods. The present data would suggest that if, for example, *ad libitum* MR was fed for 3–4 weeks of life with a subsequent gradual weaning period of 3 weeks, then growth and welfare benefits would still be realised albeit with a reduced financial investment. Whatever the length of the *ad libitum* MR fed period, the associated costs should be considered as an investment in the future of the dairy herd rather than an increase in rearing cost.

## Supporting information

S1 File Multivariable regression models for the association between morphometric measures and pre-weaning dietary group from 0 to 108 weeks, including potential confounders. Calf was included as a random effect. Coefficients for time (in weeks) and interaction terms are omitted for clarity.
(DOCX)

S2 File Marginal means (95% CI) of predicted morphometric measures by dietary group for calves from birth until 80 weeks of life. (DOCX)
