# Supplementary material for: The impact of early life nutrition and housing on growth and reproduction in dairy cattle
Source: PLoS One. 2018 Feb 14;13(2):e0191687. doi: 10.1371/journal.pone.0191687 (PMC5812595; doi:10.1371/journal.pone.0191687)
Supplement: S1 File — Calf was included as a random effect. Coefficients for time (in weeks) and interaction terms are omitted for clarity. (DOCX) [file pone.0191687.s001.docx]

**Table S.1: Multivariable regression model for the association between withers height and pre-weaning dietary group from 0 to 108 weeks, including potential confounders**. Calf was included as a random effect. Coefficients for time (in weeks) and interaction terms are omitted for clarity.

| Outcome variable: withers height | Coefficient | 95% CI | P value |
| --- | --- | --- | --- |
| *ad libitum* vs restricted MR | -0.425 | -2.044 - 1.195 | 0.607 |
| dam parity | 0.773 | -0.444 - 1.991 | 0.213 |
| plasma tp | 0.612 | -0.139 - 1.362 | 0.110 |
| pneumonia | -0.691 | -2.126 - 0.745 | 0.346 |
| diarrhoea | -0.810 | -2.124 - 0.505 | 0.227 |
| constant | 72.074 | 66.824 - 77.325 | <0.001 |

| Random-effects Parameters (variance) | Estimate | 95% CI |
| --- | --- | --- |
| calf: | 8.721 | 6.549 - 11.612 |
| Bull: | 0.727 | 0.076 - 6.974 |
| Residual | 4.255 | 4.030 - 4.492 |

**Table S.2: Multivariable regression model for the association between loin height and pre-weaning dietary group from 0 to 108 weeks, including potential confounders**. Calf was included as a random effect. Coefficients for time (in weeks) and interaction terms are omitted for clarity.

| Outcome variable: loin height | Coefficient | 95% CI | P value |
| --- | --- | --- | --- |
| *ad libitum* vs restricted MR | 0.205 | -1.312 - 1.721 | 0.791 |
| dam parity | 0.827 | -0.284 - 1.937 | 0.145 |
| plasma tp | 0.756 | 0.071 - 1.441 | 0.031 |
| pneumonia | -1.311 | -2.621 - -0.001 | 0.050 |
| diarrhoea | -1.290 | -2.489 - -0.091 | 0.035 |
| constant | 75.526 | 70.730 - 80.323 | <0.001 |

| Random-effects Parameters (variance) | Estimate | 95% CI |
| --- | --- | --- |
| calf: | 7.231 | 5.424 - 9.639 |
| Bull: | 1.73 x 10^-7^ | 3.15 x 10^-14^ - 0.944 |
| Residual | 4.384 | 4.152 - 4.628 |

**Table S.3: Multivariable regression model for the association between heart girth and pre-weaning dietary group from 0 to 108 weeks, including potential confounders**. Calf was included as a random effect. Coefficients for time (in weeks) and interaction terms are omitted for clarity.

| Outcome variable: heart girth | Coefficient | 95% CI | P value |
| --- | --- | --- | --- |
| *ad libitum* vs restricted MR | 0.053 | -2.335 - 2.441 | 0.965 |
| dam parity | 3.099 | 1.582 - 4.617 | <0.001 |
| plasma tp | 0.681 | -0.255 - 1.616 | 0.154 |
| pneumonia | -2.065 | -3.855 - -0.275 | 0.024 |
| diarrhoea | -1.055 | -2.694 - 0.585 | 0.207 |
| constant | 76.780 | 70.178 - 83.382 | <0.001 |

| Random-effects Parameters (variance) | Estimate | 95% CI |
| --- | --- | --- |
| calf: | 13.133 | 9.771 - 17.650 |
| Bull: | 1.068 | 0.044 - 26.203 |
| Residual | 18.368 | 17.398 - 19.392 |

**Table S.4: Multivariable regression model for the association between belly girth and pre-weaning dietary group from 0 to 108 weeks, including potential confounders**. Calf was included as a random effect. Coefficients for time (in weeks) and interaction terms are omitted for clarity.

| Outcome variable: belly girth | Coefficient | 95% CI | P value |
| --- | --- | --- | --- |
| *ad libitum* vs restricted MR | 1.676 | -1.676 - 5.027 | 0.327 |
| dam parity | 4.348 | 2.353 - 6.344 | <0.001 |
| plasma tp | 1.342 | 0.112 - 2.572 | 0.033 |
| pneumonia | -2.597 | -4.951 - -0.244 | 0.031 |
| diarrhoea | -2.182 | -4.338 - -0.027 | 0.047 |
| constant | 74.485 | 65.770 - 83.200 | <0.001 |

| Random-effects Parameters (variance) | Estimate | 95% CI |
| --- | --- | --- |
| calf: | 22.335 | 16.537 - 30.165 |
| Bull: | 4.924 | 1.231 - 19.697 |
| Residual | 41.681 | 39.480 - 44.004 |

**Table S.5: Multivariable regression model for the association between crown to rump length and pre-weaning dietary group from 0 to 108 weeks, including potential confounders**. Calf was included as a random effect. Coefficients for time (in weeks) and interaction terms are omitted for clarity.

| Outcome variable: crl | Coefficient | 95% CI | P value |
| --- | --- | --- | --- |
| *ad libitum* vs restricted MR | 0.773 | -2.134 - 3.680 | 0.602 |
| dam parity | 1.573 | -0.190 - 3.336 | 0.080 |
| plasma tp | 0.837 | -0.250 - 1.924 | 0.131 |
| pneumonia | -2.157 | -4.237 - -0.078 | 0.042 |
| diarrhoea | -2.463 | -4.367 - -0.558 | 0.011 |
| constant | 78.959 | 71.268 - 86.650 | <0.001 |

| Random-effects Parameters (variance) | Estimate | 95% CI |
| --- | --- | --- |
| calf: | 17.520 | 12.995 - 23.621 |
| Bull: | 4.06 x 10^-10^ | 2.95 x 10^-17^ - 0.006 |
| Residual | 30.246 | 28.649 - 31.932 |

**Table S.6: Multivariable regression model for the association between hock-fetlock length and pre-weaning dietary group from 0 to 108 weeks, including potential confounders**. Calf was included as a random effect. Coefficients for time (in weeks) and interaction terms are omitted for clarity.

| Outcome variable: hock-fetlock length | Coefficient | 95% CI | P value |
| --- | --- | --- | --- |
| *ad libitum* vs restricted MR | -0.334 | -0.960 - 0.291 | 0.295 |
| dam parity | 0.355 | -0.026 - 0.737 | 0.068 |
| plasma tp | 0.267 | 0.032 - 0.503 | 0.026 |
| pneumonia | -0.035 | -0.485 - 0.415 | 0.880 |
| diarrhoea | -0.478 | -0.890 - -0.065 | 0.023 |
| constant | 33.454 | 31.789 - 35.119 | <0.001 |

| Random-effects Parameters (variance) | Estimate | 95% CI |
| --- | --- | --- |
| calf: | 0.823 | 0.611 - 1.109 |
| Bull: | 9.96 x 10^-18^ | 1.07 x 10^-26^ - 9.26 x 10^-9^ |
| Residual | 1.384 | 1.311 - 1.461 |

**Table S.7: Multivariable regression model for the association between body condition score and pre-weaning dietary group from 0 to 108 weeks, including potential confounders**. Calf was included as a random effect. Coefficients for time (in weeks) and interaction terms are omitted for clarity.

| Outcome variable: bcs | Coefficient | 95% CI | P value |
| --- | --- | --- | --- |
| *ad libitum* vs restricted MR | 0.085 | -0.034 - 0.204 | 0.160 |
| dam parity | 0.024 | -0.040 - 0.087 | 0.462 |
| plasma tp | 0.052 | 0.013 - 0.091 | 0.009 |
| pneumonia | -0.074 | -0.148 - 0.001 | 0.054 |
| diarrhoea | 0.010 | -0.058 - 0.079 | 0.773 |
| constant | 2.415 | 2.137 - 2.693 | <0.001 |

| Random-effects Parameters (variance) | Estimate | 95% CI |
| --- | --- | --- |
| calf: | 0.022 | 0.016 - 0.030 |
| Bull: | 0.009 | 0.003 - 0.027 |
| Residual | 0.051 | 0.049 - 0.054 |
