# Supplementary material for: The impact of early life nutrition and housing on growth and reproduction in dairy cattle
Source: PLoS One. 2018 Feb 14;13(2):e0191687. doi: 10.1371/journal.pone.0191687 (PMC5812595; doi:10.1371/journal.pone.0191687)
Supplement: S2 File — (DOCX) [file pone.0191687.s002.docx]

**b**

**a**

**d**

**c**

**Figure S.1:** Marginal means (95% CI) of a) predicted body weight (kg), b) predicted BCS, c) predicted heart girth (cm) and d) predicted belly girth (cm) for calves in Group A (blue line) and R (red line) from birth until 80 weeks of age.

**a**

**d**

**c**

**b**

**Figure S.2:** Marginal means (95% CI) of a) predicted withers height (cm), b) predicted loin height (cm), c) predicted crown rump length (cm) and d) predicted hock-fetlock length (cm) for calves in Group A (blue line) and R (red line) from birth until 80 weeks of life.
